# Supplementary material for: The chlamydial transcriptional regulator Euo is a key switch in cell form developmental progression but is not involved in the committed step to the formation of the infectious form
Source: mSphere. 2024 Aug 14;9(9):e00437-24. doi: 10.1128/msphere.00437-24 (PMC11423577; doi:10.1128/msphere.00437-24)
Supplement: Supplemental figures — Fig. S1 through S4; movie legends. [file msphere.00437-24-s0001.pdf]

Supplemental Figures:

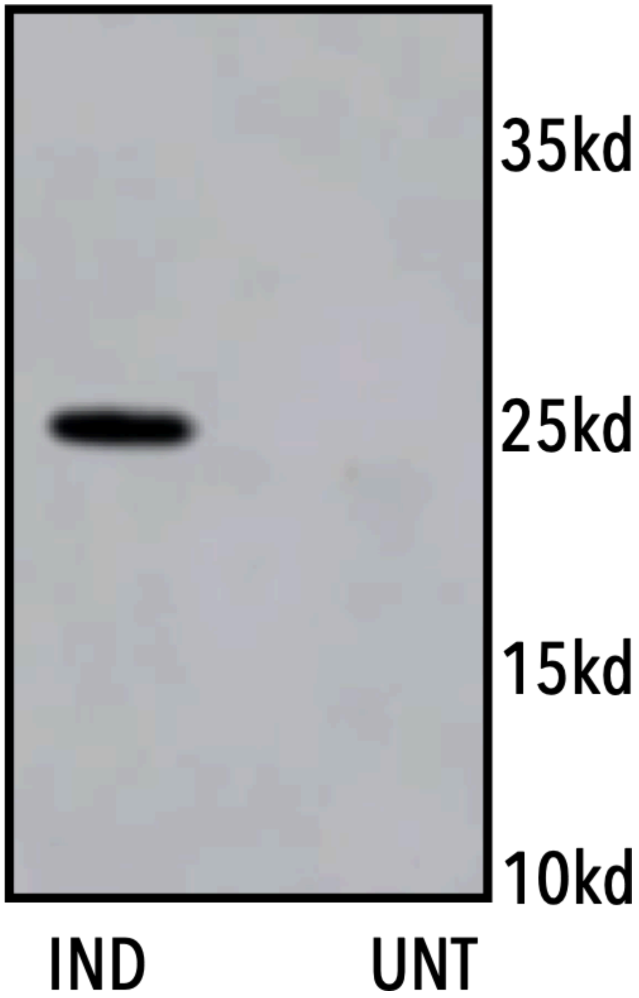

Figure S1: Western blot of Euo-FLAG. Anti-FLAG western blot of Cos-7 cells infected with L2-E-Euo-FLAG comparing Euo-FLAG expression in Tph treated and untreated cultures. Cells were induced or not with 0.5mM Tph at 16 hpi and proteins were harvested at 30 hpi, separated by PAGE, transferred to a nitrocellulose membrane and probed for the presence of the FLAG tag.

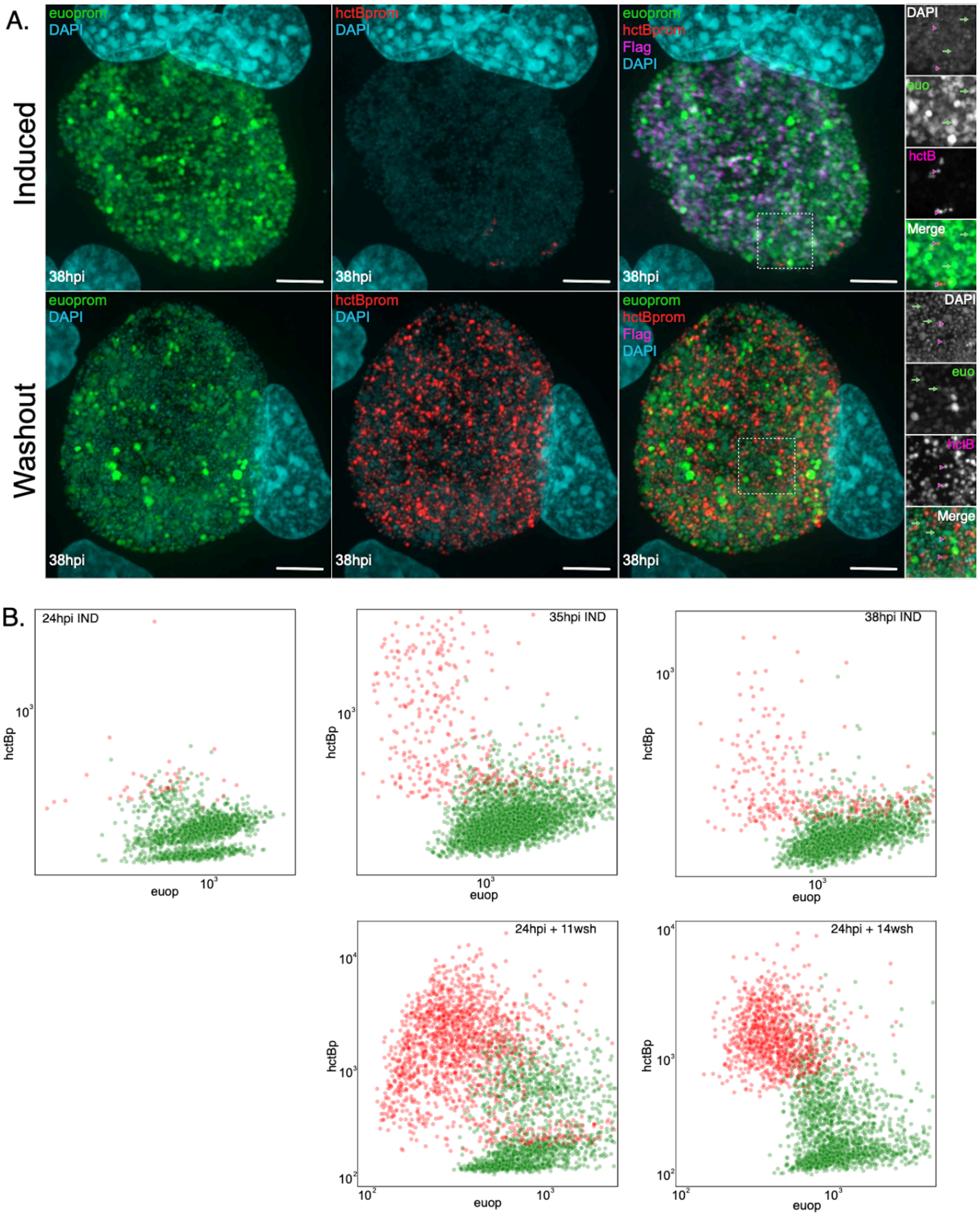

Figure S2: Reentry into the developmental cycle after inducer washout. A) Confocal micrographs of Cos-7 cells infected with L2-E-Euo-BmeC induced with 0.5mM TPH at infection. The 38 hpi inclusions in the induced control infected cells were imaged using confocal microscopy and had primarily *euo*prom+ cells (green) with few *hctB*prom+ cells (red). These cells were stained positive for Euo-FLAG (magenta). Tph was washed out at 24 hpi and the cells were fixed 14 hours later at 38 hpi, stained for Euo-FLAG expression and imaged using confocal microscopy. The washout inclusions had fewer *euo*prom+ cells (green) and an increase in *hctB*prom+ cells (red). These cells had essentially no staining for Euo-FLAG (megenta). Scale bar =15 µm. B) Cells were infected with L2-E-Euo-BmeC and induced with 0.5mM TPH at infection. Individual *euo*prom+ cells (green) and *hctB*prom+ cells (red) were identified using the Trackmate in FIJI and the fluorescence signal

for both *euo*prom and *hctB*prom in individual chlamydial cells from five inclusions was determined. These values were plotted for each cell, green (*euo*prom+) and red (*hctB*prom+). At 24 hpi the inclusions contained primarily *euo*prom+ (green) fluorescent signal. Tph was removed, fluorescent intensity was determined for both *euo*prom+ (green) and *hctB*prom+ (red) cells at 11 and 14 hours post washout (hpw) and plotted. Red cells positive for *hctB*prom signal increased over time after washout.

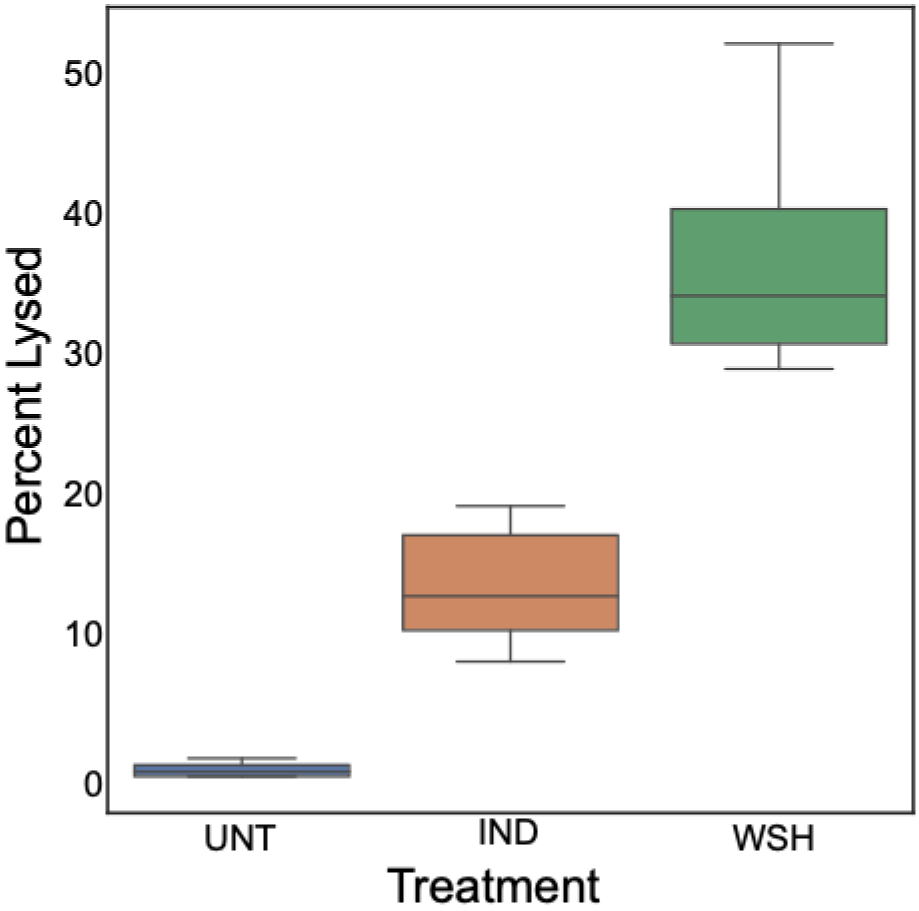

Figure S3. Quantification of inclusion/cell lysis after Tph washout. Cos-7 cells were infected with L2-E-Euo-BmEc and treated with Tph or vehicle only at infection. At 24 hpi Tph was washed out and the infected cultures were imaged every 30 minutes for expression of GFP (*euo*prom) and RFP (*hctB*prom). At 3 hours post washout the number of inclusions that visibly lysed was quantified for the untreated (UNT), Tph induced (IND) and Tph washout (WSH) cultures. Images were taken at 4x magnification. 8 FOV were counted per treatment with n > 100 inclusions counted per FOV.

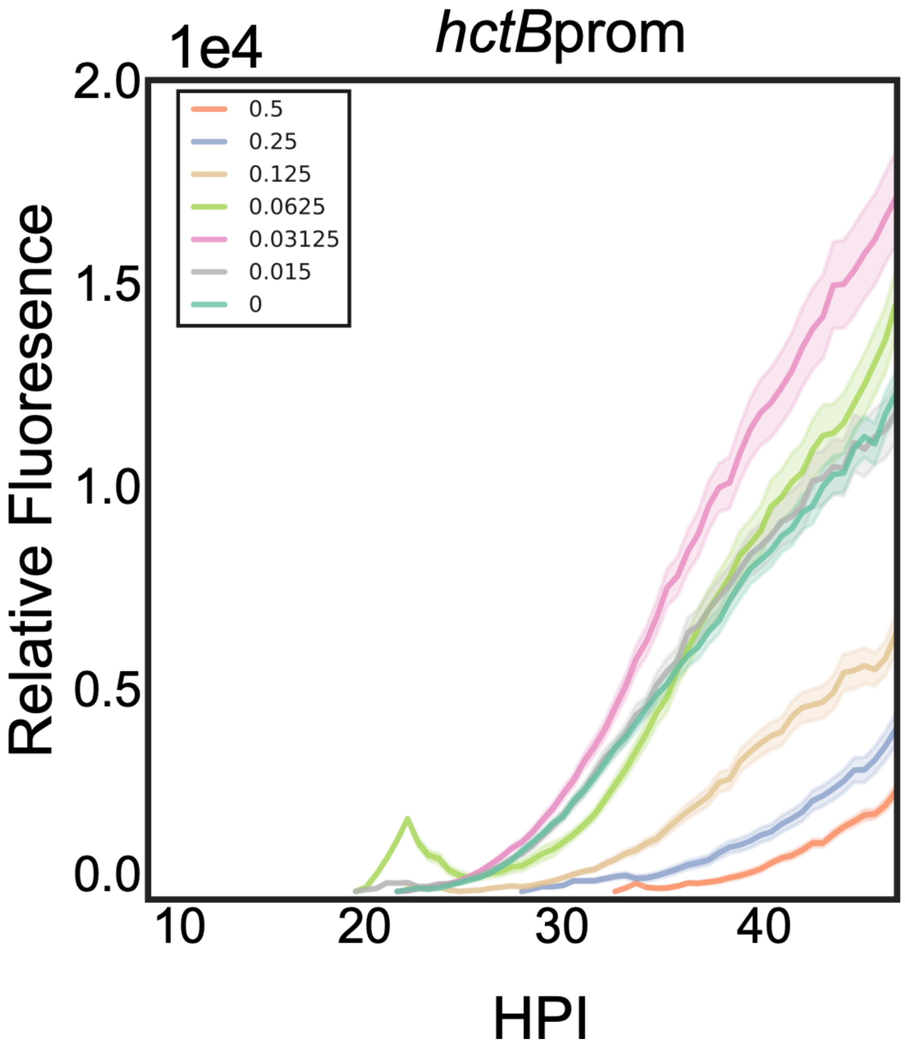

Figure S4: Kinetics of the developmental cycle of L2-euonprom-E-Euo-Bng induced for Euo expression with a range of Tph. Cos-7 cells infected with L2-*euo*nprom-E-Euo-Bng and were induced for Euo expression at 0 hpi with 0.5, 0.25, 0.125, 0.0625, 0.03125, 0.015 or Tph or vehicle only and assayed for *hctB*prom activity by live cell microscopy. Error cloud for fluorescent reporter represents SEM. n > 20 inclusions per treatment.

Supplemental Movie 1: Cos-7 cells infected with E-Euo-BmEc and induced for Euo-FLAG expression at infection with 0.5 mM Tph. At 24 hpi the infected cells were imaged every 30 minutes for expression of GFP (*euo*prom) and RFP (*hctB*prom) for an additional 56 hours.

Supplemental Movie 2: Cos-7 cells infected with E-Euo-BmEc and induced for Euo-FLAG expression at infection with 0.5 mM Tph. At 24 hpi Tph was removed and the infected cells were imaged every 30 minutes for expression of GFP (*euo*prom) and RFP (*hctB*prom) for an additional 56 hours.
