# Supplementary material for: The chlamydial transcriptional regulator Euo is a key switch in cell form developmental progression but is not involved in the committed step to the formation of the infectious form
Source: mSphere. 2024 Aug 14;9(9):e00437-24. doi: 10.1128/msphere.00437-24 (PMC11423577; doi:10.1128/msphere.00437-24)
Supplement: Supplemental tables — Tables S1 and S2. [file msphere.00437-24-s0002.pdf]

Supplemental Tables:

Construct/Use	Primer Name	Template
**E-Euo_3xFlag (cb)**		
GATGGTGAGCgaatgcttacaacaagatacaggggtc	5' Euo Estar	**L2 Genomic**
TGTAGTCCATtgagataaaattttctgcgtctgccag	3' Euo Estar	**L2 Genomic**
ttttatctcaATGGACTACAAAGACCATGACGGTG	5' Estar_vector Euo	**p2TK2_E-Clover-3xFlag**
GTAAGCATTCgctcaccatcttgttgttacctcc	3' Estar_vector Euo	**p2TK2_E-Clover-3xFlag**
**E-Euo_3xFlag (spec)**		
gtagtcggcaaaTAACTGTCAGACCAAGTTTACTCATATATAC	5' ptK2-sw-hctA-clover	**pUC18A**
tctggaccagttgcgtgagcgcatCATTGGAAAACGTTCTTCGGGGCGAAAAC	3' ptK2-sw-hctA-clover	**pUC18A**
atgcgctcacgcaactggtccagaACCTTGACCGAACGCAGCGGTG	5' aadA (spec) from pBam4	**p2TK2_E-Euo-3xFlag (cb)**
ttggtctgacagTTATTTGCCGACTACCTTGGTGATCTCG	3' aadA (spec) from pBam4	**p2TK2_E-Euo-3xFlag (cb)**
**p2TK2_E-Euo-3xFlag_HctBp-mKate2_EUOp-Clover (spec) (BmeC)**		
gtttttaacATTTTTTACGGTTCCTGGCCTTTTGCTGGCCTTTTGC	Flag 5' (HctBp_Euop)	**p2TK2_E-Euo-3xFlag (spec)**
gtacaagTAGTGGCCGCGTTGCTGGCGTTTTTCCATAGG	Flag 3' (HctAp_IhtAp)	**p2TK2_E-Euo-3xFlag (spec)**
CCGTAAAAAATgttaaaaactaaccatttttattaaagtttttcattctccttgtcg	HctBp_EUOp 3' (Flag)	**p2TK2_hctBprom-mkate2_euoprom-mClover (BmeC)**
AACGCGGCCACTActtgtacagctcgtccatgccatgtgtaatcc	HctAp_IhtAp 5' (Flag)	**p2TK2_hctBprom-mkate2_euoprom-mClover (BmeC)**
**p2TK2-EuoNativeProm-riboj-E-EuoCodonOpt_3xFlag**		
CGGTTATCCACAGAATCAGGGGATAACGCAGG	EuoNativeProm-BB 3'	**p2TK2_E-Euo-3xFlag (cb)**
atgGACTACAAAGACCATGACGGTGATTATAAAGATCATGACATCG	EuoNativeProm-BB 5'	**p2TK2_E-Euo-3xFlag (cb)**

CCTGATTCTGTGGATAACCGTATTACATtattttt aacaaaccacttgattaataagttttttgttgggaaaatattacct tctctttttaaggattttgcaatttttcagtaagcgctcgctaaacta ggaagagaaagttatgaatagagtggaaagggcttgtccga cttagagattcaataagcatagctctaagagacggggttaga aggtcacagagccattattcacaagacaggccaagatttgtttt taagtggacgagagaggactaaacagtcgtagctgtcaccg gatgtgctttccggtctgatgagtccgtgaggacgaaacagcc tctacaaataattttgtttaaGGTGATACCAGCATCGT CTTGATGCCCTTGGCAGCACCCTGCTAAG GAGGTAACAACAAGATGGAGTGCTTACAAC AGGACACGGGCGTGGAGGCTGAGCAAGT ACAAGTCCAACAACAAGAGGAAAACGCAG TCCCAGTGACGAGTCAAAGAGTGTCCATAA CTCAAGCTGCCAAGTTACACAATGTTACAA GACAAGCAATATACGTCGCTATAAAGCAGA AGAAACTCAAAGCTTCTAAAACCACTAGAT GGGAAATAGACCTTCAAGATTTGGAGGATT ACAGACGTAATCGTTACTCACGCGCGAAGT CTACCTACCAGGGAGAACTACTATTTGACA ACGAGAAAGGTTTTTATTCCGTGGGGCAG GTGGCCTCTATGCTCGATGTCCCAGAACAA AAAATTTACTATGCAACCCGTATAGGCGCAA TGAAAGGAGAGAGACGCGGGTCCGCTTG GGTAATCCACGTTTCCGAGGTAGACCGCTA CCGTAATGATTACTTGAAGAAAGAAGCAGA GAGAAAAGGTAAATCCTTAGCCGCAATGCG CGAGGGTTTTGAGGCGCTAGGTGCCGACC TACTTGCAGATGCGGAAAATTTTATTTCAatg GACTACAAAGACCATGA		**EuoProm-J-E-Euo (codon substituted)-Flag_gblock**
**p2TK2-EuoNativeProm-riboj-E-EuoCodonOpt_3xFlag**		
GTTGCTGGCGTTTTTCCATAGGCTCC	3' ngsynterm bb	**p2TK2-EuoNativeProm-riboj-E-EuoCodonOpt_3xFlag**
acATTTTTTACGGTTCCTGGCCTTTTGCTGG	5' ngsynterm bb	**p2TK2-EuoNativeProm-riboj-E-EuoCodonOpt_3xFlag**

TATGGAAAAACGCCAGCAACGCGGCCAggacatgcgatcgtattgcctatgaggaccaaaacgaaaaaggccccccctttcgggaggcctcttttctggaatttggtaccgagctatctaaggatggagggccaaggatgcagacgtaaaaaaagcggcgtggttagccgctttttaattgccggagatccttactatttatacagttcatccatgcccatcacatcggtaaacgctttctgccattctttaaagttcagttcggttttgctatgtttcagttcggttttgcgaaacacatacatcggctggtttttcagatagttcgccgccatcggtttcgcaaaggtataggtggtgcgcgcggtgctgcgatagcgtttgccgttgccggtggtatagctccatttaaaggtgctaataatggttttatcgttcggataggtttttttgctgcggcaccaatccgccgcggtcaggctgttggtcatcaccgggccatccgccggaaagccggtgcctttcacctgcgcttcgcctttaatatggctgccttcataggtatagcgatagttcacggtcaggctcgcgccatcttcaaactgcatggtgcgatgcacctgatagccgctgccatccaccatcgccgcctgaaacgggctcatgccatccggatacggcagatactgatgaaagccatagccaatatgcggcaccagaatccacgggctaaactgcagatcgcctttggtgcttttcaggttcagttcttcatagccatcgttcgggttgccggtgccctggcccaccatatcaaaatccacgccgttaatgctgccaaaaatatgcagttcatgggtcgccggcaggctcgccatgttatcttcttcgcctttgctcaccatgccgaattcGCGTTTCTTTTGTACTCCCAACATGTTCATtcccctaattagacaggtaactactacttatttgatctatcgacaaggagaatgaaaaactttaataaaaaatggttagttttttaacATTTTTTACGGTTCCTGGCCTTTTGCT		**hctBneongreen-synterm_gblock**

Supplemental Table 1: Plasmid and primer table

Gene	Seq Length	Seq Range
euo	739	203142 - 203880
hctA	561	895571 - 896131
Tarp	3072	849421 - 852492
IncD-G	1676	457415 - 459091

Supplemental Table 2:In-Situ Probes
